# Goldenhar syndrome with limbal neoformation, microtia and skeletal deformities: a case report and literature review

**DOI:** 10.1186/s12886-024-03317-9

**Published:** 2024-02-22

**Authors:** Yushan Fu, Haotian Yu, Jiajia Zhang, Nan Zhou

**Affiliations:** Department of Ophthalmology, the Second Affiliated Hospital of Harbin Medical University, Harbin, 150086 China

**Keywords:** Goldenhar syndrome, Hemifacial microsomia, Mandibular hypoplasia, Epibulbar dermoid

## Abstract

**Background:**

To report a case of a 4-year-old patient with Goldenhar syndrome.

**Case presentation:**

The author presents a rare case report involving a 4-year-old boy with multiple malformations. A comprehensive examination showed that the patient primarily had a limbal dermoid. He also has bilateral microtia and ear canal deformities. The skull CT scan and spine X-ray showed Maxillofacial Abnormalities and scoliosis. Whole Exome Sequencing revealed potential gene variations related to microtia. Although certain circumstances prevented us from initiating follow-up treatment for the patient, we have provided a detailed account of the diagnostic methodologies used for this condition.

**Conclusions:**

Goldenhar syndrome is a congenital condition, predominantly presenting as sporadic cases. Its diagnosis and management typically necessitate the involvement of multiple disciplines, including otolaryngology and craniofacial surgery. The syndrome encompasses a variety of craniofacial features, which can facilitate early diagnosis and guide subsequent therapeutic interventions.

## Background

Goldenhar syndrome (GS), alternatively referred to as Oculo-auriculo-vertebral syndrome (OAVS), is an innate anomaly [1]. The first case of GS was reported in the 1950 by the French ophthalmologist Maurice Goldenhar [2]. Now it affects one in every 3,000–5,000 births, the ratio of male to female incidence 3:2 [3].

This syndrome can cause a diverse range of oral and systemic manifestations, with the severity of abnormalities and symptoms varying from individual to individual. The classical manifestations of this condition encompass the absence or partial development of the auricles, coupled with impairments in the middle or inner ear, culminating in auditory impairment. Furthermore, prevalent characteristics comprise hemifacial microsomia, as well as hypoplasia of the maxilla or mandible. Congenital scoliosis, found in approximately 50% of cases, and epibulbar dermoids posing risks to visual acuity are also recurrent features. Notably, Goldenhar syndrome may extend its impact to visceral organs, encompassing the cardiac, renal, and nervous systems.

Goldenhar syndrome (GS) manifests with a wide array of clinical manifestations. However, the primary approach to diagnosis revolves around the triad of mandibular hypoplasia accompanied by facial asymmetry, oculo-auricular malformations, and vertebral abnormalities. Additional diagnostic tests may be employed in conjunction with the aforementioned criteria to further support the accurate identification of GS [2, 4, 5].

This article presents a case report of Goldenhar syndrome in a 4-year-old male patient. The patient shows symptoms affecting multiple organ systems. These include an epibulbar dermoid in the eyes, significant defect in the mandible, microtia, preauricular tags, external auditory canal deformity, and scoliosis. The case’s phenotypic presentation is both typical and comprehensive, making it incredibly rare. Notably, the patient primarily sought medical assistance at the Ophthalmology Clinic for an inferior temporal limbal neoformation. This highlights the critical importance of ophthalmologists having knowledge of relevant syndromes for accurate and timely diagnosis.

## Case presentation

A 4-year-old male patient presented at the Ophthalmology Clinic of the Second Affiliated Hospital of Harbin Medical University, with the chief complaint of inferior temporal limbal neoformation on the left eye. According to the patient’s parents, the lesion was congenital, and had slowly and progressively grown throughout the patient’s life. The parent reported the absence of any other ocular complaints, history of ocular trauma, concurrent systemic illnesses, or the use of systemic or topical medications in the medical history. There was no history of consanguinity, and no abnormalities were noted in the parent. However, the patient’s mother had diabetes mellitus before conceiving and did not take any measures to intervene.

**Fig. 1:**
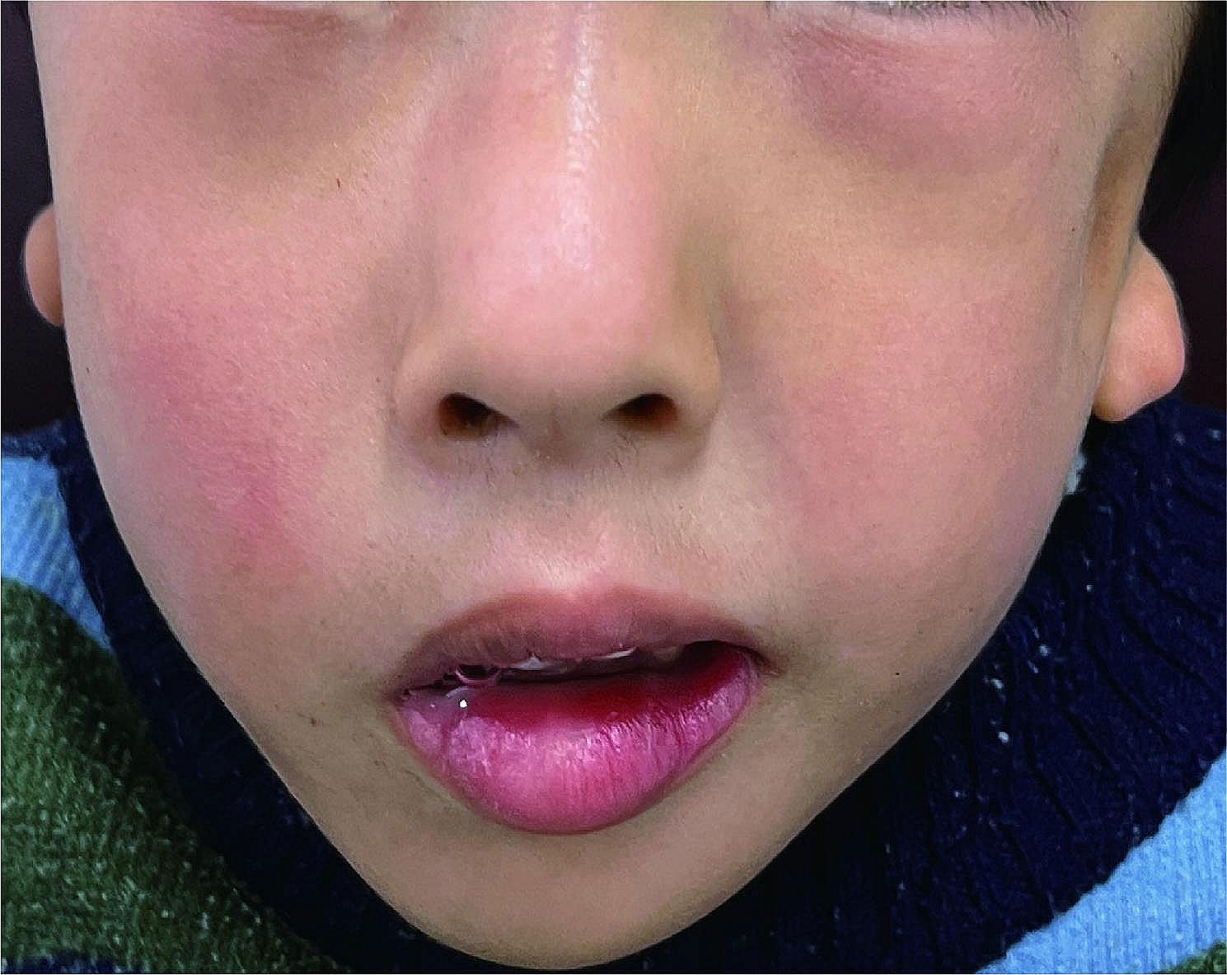
The asymmetry of the patient’s face

**Fig. 2:**
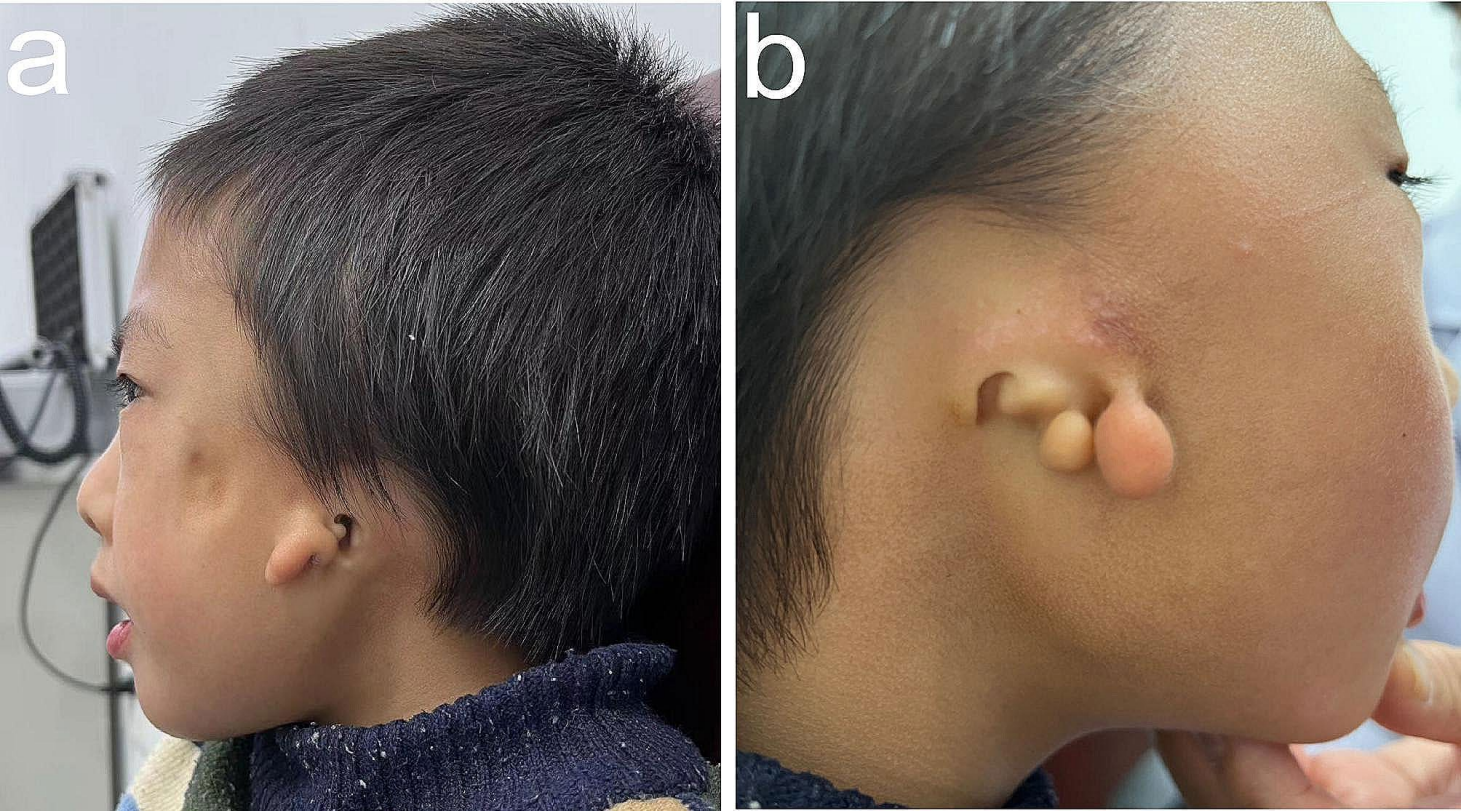
Microtia and preauricular tags on both sides. (**a**) The patient exhibits narrowness of the left external auditory canal. (**b**) The patient demonstrates absence of the right external auditory canal

Upon examination, the patient exhibited facial asymmetry, characterized by a leftward deviation of the angle of the mouth (Fig. 1). Notably, there was a reduced prominence of the left malar area, resulting in flattening of the left side of the face. Furthermore, the facial profile was concave due to midface deficiency. The patient also had mild mandibular hypoplasia and obvious malformation of both external ears. Microtia and preauricular tags were present on both sides (Fig. 2). However, a narrow external aural was observed only in the left ear, with the right external aural missing.

**Fig. 3:**
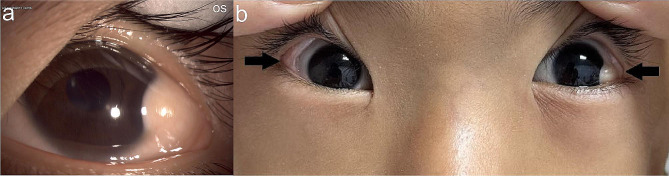
Epibulbar dermoid and bilateral lipodermoids of the patient’s eyes. (**a**) Ophthalmic anterior segment Imaging of the patient showing epibulbar dermoid in the left eye. (**b**) The black arrow indicates the bilateral lipodermoids of the patient’s eyes

**Fig. 4:**
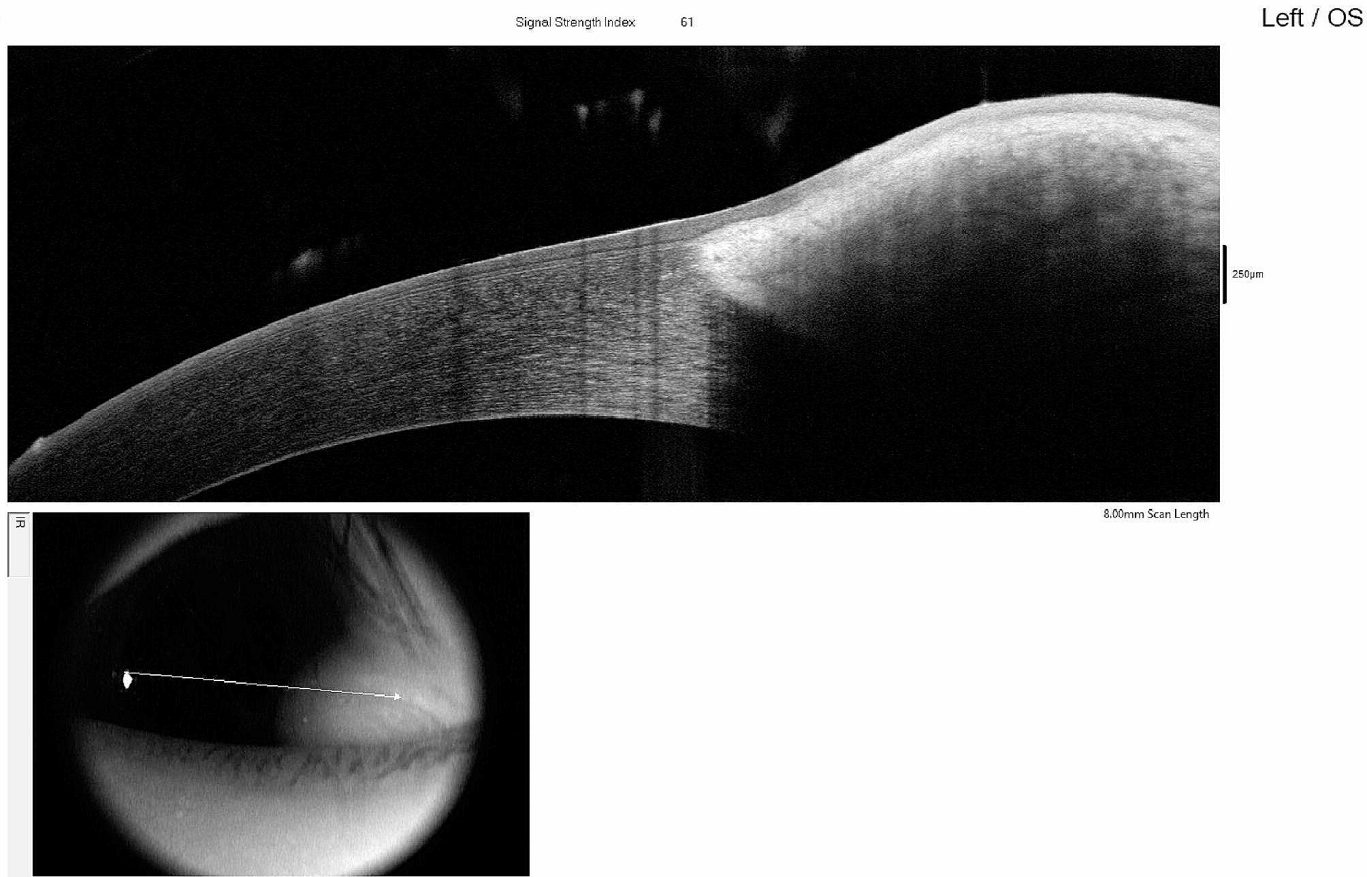
OCT show the depth of the epibulbar dermoid infiltration into the stroma

The patient had hypertelorism with an epibulbar dermoid in the left eye (Fig. 3a) and bilateral lipodermoids (Fig. 3b). Biomicroscopy of the right eye showed no abnormalities, while the left eye exhibited a nodular, elevated lesion located at the inferior temporal corneal-limbo-conjunctival area, with deep infiltration into the stroma (Fig. 4). No abnormalities were detected in any of the other ophthalmological examinations. In addition, an intraoral examination revealed multiple carious teeth, malocclusion, and left posterior crossbite. Subsequently, the patient underwent radiographic evaluation.

**Fig. 5:**
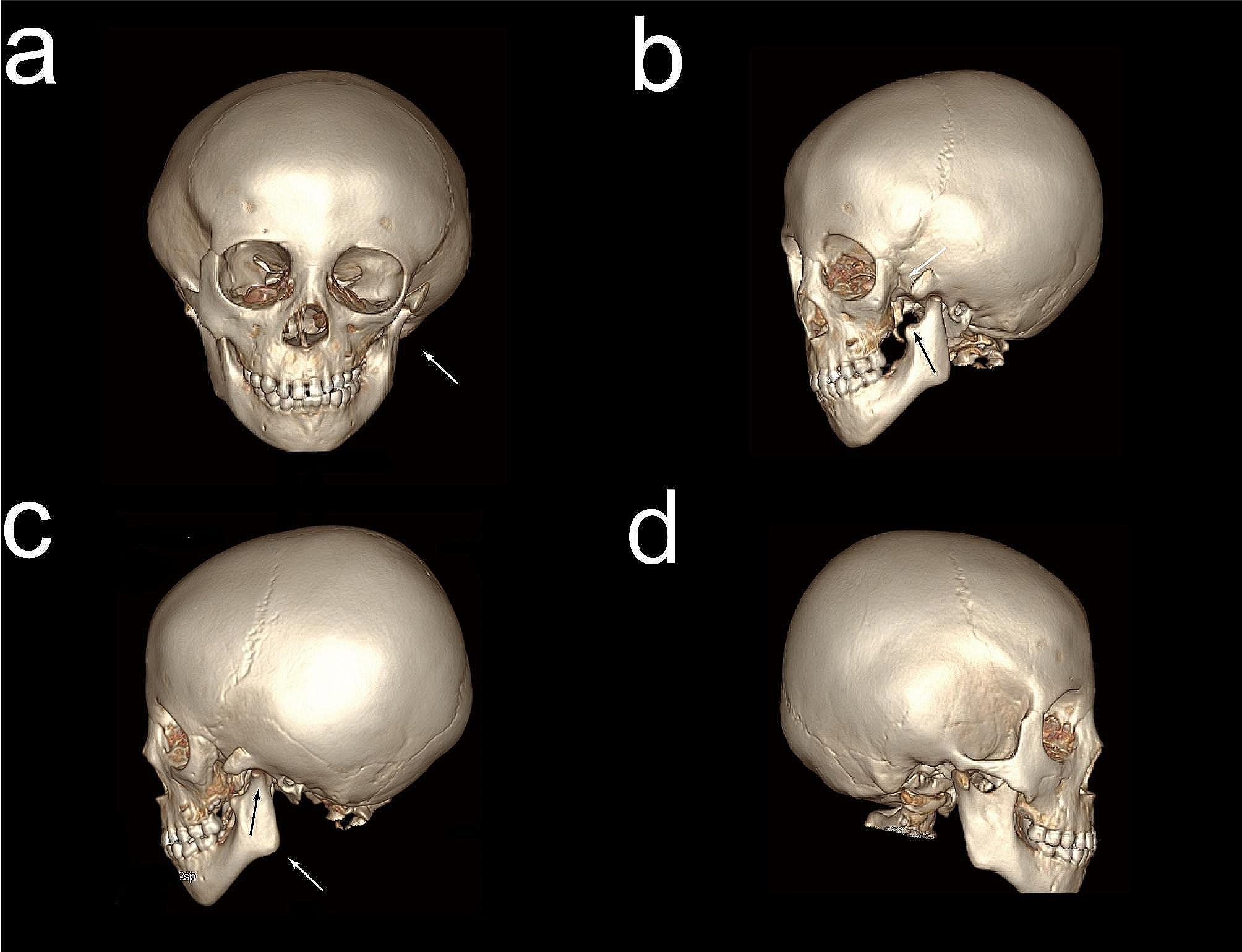
The skull CT scan of the patient. (**a**) Skull CT scan (white arrow) showed asymmetry of the bilateral mandible and zygomatic bone. (**b**) The left zygomatic bone lacks a temporal process (white arrow). And there is agenesis of the coronoid process of the mandible (black arrow). (**c**) Hypoplasia of the mandibular (white arrow) and the condyle (black arrow) on the left side. (**d**) CT scan of normal skull on the right side

**Fig. 6:**
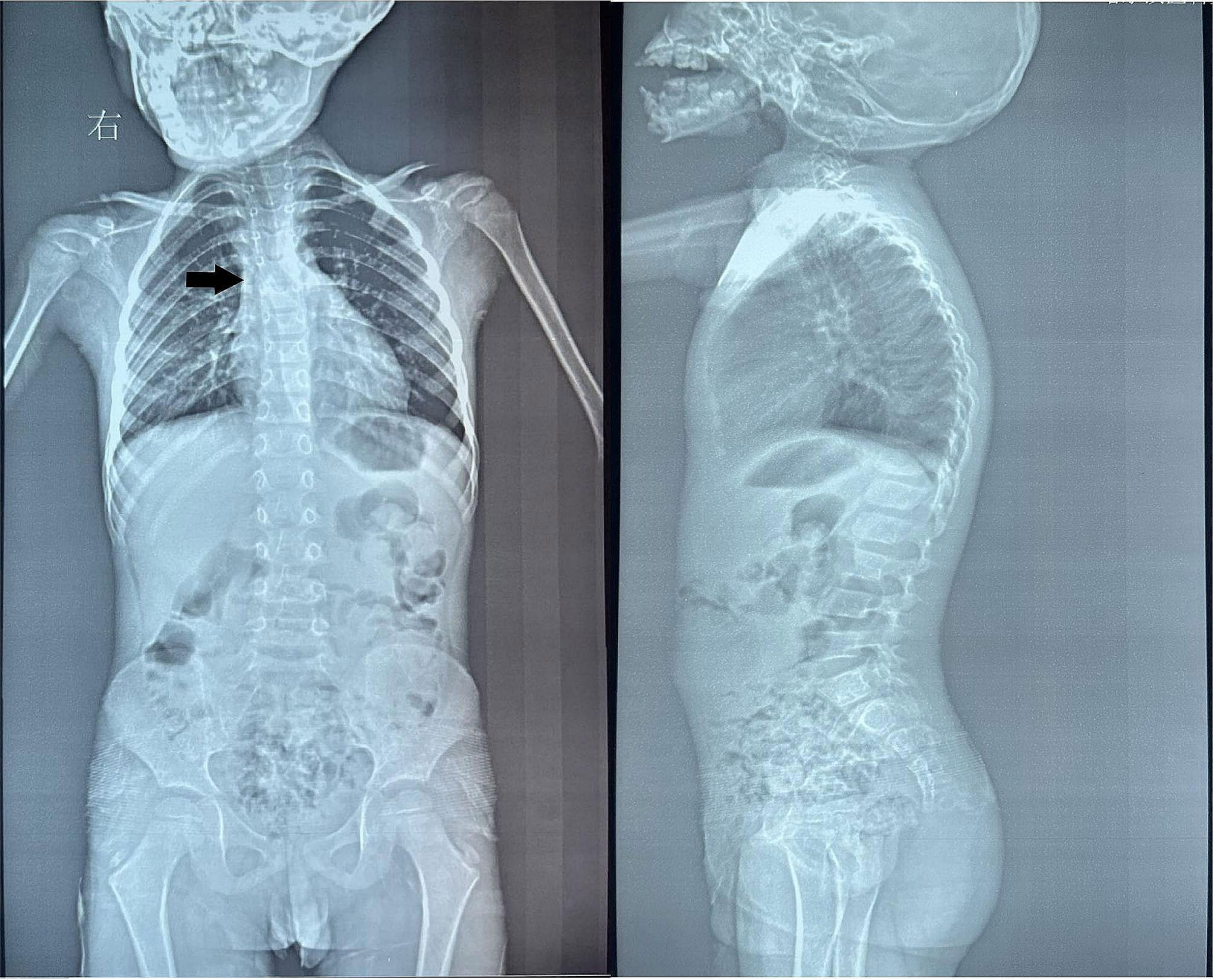
The spine radiograph (black arrow) showing scoliotic spine

Orthopantomography revealed no missing teeth and good tooth germ development. A Skull CT scan showed asymmetry of the bilateral mandible and zygomatic bone. The left zygomatic bone lacks a temporal process. Agenesis of the coronoid process of the mandible, along with mandibular hypoplasia and left-sided condylar hypoplasia, can be observed in Fig. 5. The spine radiograph revealed a slight curvature to the left side (Fig. 6). GS is often associated with cardiac or renal hypoplasia. To assess this, we arranged for an ultrasound of heart and kidneys, which showed no obvious dysplasia. Furthermore, the patient’s EKG, blood routine examination and blood biochemical tests were normal.

In this study we sequenced the patient’s Whole Exome Sequencing (WES) and found 5 gene variations that may be related to microtia (Table 1). Based on the clinical findings and radiographic features, the patient was diagnosed with Goldenhar syndrome.

**Table 1:** The potential gene variations related to microtia

Gene	Variation information
PRDM16	Chr1:3328445 c.1684G > A p.V562I
FAT4	Chr4:126412906 c.14935G > A p.A4979T rs17009858
POLR1C	Chr6:43487568 c.374G > A p.R125Q
PLEC	Chr8:144993545 c.10,444 C > T p.R3482C rs370181826
MED12	ChrX:70,340,926 c.659G > A p.G220E

## Discussion and conclusions

The OAVS arises due to abnormal embryonic development of derivatives originating from the first and second branchial arches. Despite extensive research, the etiology of OAVS largely remains elusive.

Several chromosomal anomalies, such as mosaic and/or partial trisomies, as well as copy number variants (CNVs), have been identified as potential contributors to this intricate disorder. Additionally, prenatal environmental factors have been hypothesized as potential causal agents for Goldenhar syndrome [6].Maternal and fetal hypoxia, maternal hypertension, maternal diabetes, viral infections such as influenza or rubella, malnutrition, smoking, exposure to ionizing radiation during the pregnancy through radio-diagnostic procedures, as well as exposure to certain drugs like cocaine, tamoxifen, ibuprofen, and retinoic acid, and consanguineous marriage have been identified as potential predisposing factors for Goldenhar syndrome [4, 5, 7–9].

Moreover, twin pregnancies have been associated with an elevated risk of exhibiting the OAVS phenotype. It is noteworthy that twin patients with OAVS can present both concordant and discordant phenotypes, even in cases of monozygotic twins [10–12]. However, in most cases, the syndrome is sporadic [2, 5, 7].

The term “OAVS” (Oculo-Auriculo-Vertebral Syndrome) was coined by Robert Gorlin in 1963 to refer to the full malformation association. Prior to the adoption of the terminology Goldenhar syndrome, alternative synonyms used to describe this condition included facio-auriculo-vertebral syndrome and Goldenhar Gorlin syndrome. Hence, the spectrum of symptoms observed and their respective severity can exhibit considerable variation among affected individuals.

Goldenhar syndrome commonly presents with various eye symptoms including epibulbar dermoids, microphthalmia, strabismus, cataracts, coloboma of the iris and retina, as well as coloboma of the upper eyelid. Among ear changes, it is possible to observe auricular appendage, asymmetry of the ears, atresia of the auditory external canal, and preauricular fistulas. In addition to the eye symptoms mentioned earlier, Goldenhar syndrome individuals may also present with hemifacial microsomia, facial asymmetry, mandibular and/or maxillary hypoplasia, dental alterations such as supernumerary teeth, Fallot’s tetralogy, dextrocardia, transposition of the great vessels, gastrointestinal tract atresia, scoliosis, microcephaly, hydrocephalus, hypoplasia of the corpus callosum, and several other associated abnormalities [2, 4, 8]. Children affected by Goldenhar syndrome may also exhibit characteristics such as low height, delayed psychomotor development, intellectual disability, speech disorders, psychosocial challenges, as well as autistic behaviors. These additional features can contribute to the complex clinical presentation and overall impact on the affected individuals [13].

Due to the varying scopes of clinical phenotypes, there are currently no established guidelines for the minimum diagnostic criteria for GS [2].

The patient in this case demonstrated typical manifestations of Goldenhar syndrome, including the classic triad of features: mandibular hypoplasia with facial asymmetry, vertebral changes, and ocular-auricular abnormalities. Specifically, the ocular-auricular changes observed in the patient encompassed the presence of a dermoid tumor of the cornea and absence of outer ear formation. These findings further support the diagnosis of Goldenhar syndrome in the patient. Although the genetic test did not reveal any obvious abnormalities, the patient’s typical clinical manifestations are clear for the diagnosis of GS.

Treatment of Goldenhar syndrome varies greatly depending on the needs of the individual. It requires a multistage and multidisciplinary approach. Therapy typically starts early and is long-lasting.

Otolaryngological consultation plays a crucial role in the diagnosis and management of patients with Goldenhar syndrome, as this condition is frequently associated with hearing loss. Given the potential involvement of the ear and related structures in Goldenhar syndrome, an otolaryngologist can conduct comprehensive evaluations, including audiological assessments, to determine the nature and extent of hearing impairment. This information is invaluable in guiding appropriate intervention strategies and ensuring optimal communication and auditory outcomes for individuals with Goldenhar syndrome.

Ophthalmological consultation is an essential component of diagnosing children with Goldenhar syndrome. Since ocular abnormalities are common in this condition, a comprehensive ophthalmic evaluation is necessary to identify and assess specific conditions such as epibulbar dermoids, dermoid cysts on the cornea and/or sclera, and coloboma. Surgical intervention may be required to address these issues, as they have the potential to impact the patient’s vision and overall eye health. Early detection and appropriate management can help mitigate potential vision problems and optimize visual outcomes for individuals with Goldenhar syndrome.

Furthermore, the physical appearance of patients with Goldenhar syndrome and their potential experiences of social rejection can contribute to the development of psychiatric disorders. These psychological challenges may warrant the involvement of psychiatric consultation and therapy. By addressing the emotional well-being of individuals with Goldenhar syndrome, psychiatric interventions can help improve their overall quality of life and promote mental health [2].

## Data Availability

Not applicable.

## Abbreviations

GS: Goldenhar syndrome
OAVS: Oculo-auriculo-vertebral syndrome
WES: Whole Exome Sequencing
CNVs: Copy number variants
